# Efficacy comparison of oxcarbazepine and levetiracetam monotherapy among patients with newly diagnosed focal epilepsy in China: A multicenter, open‐label, randomized study

**DOI:** 10.1111/cns.13840

**Published:** 2022-04-15

**Authors:** Haoyue Zhu, Xuejun Deng, Li Feng, Yajun Lian, Xiong Han, Zhenli Guo, Yulan Gou, Yuanmin Du, Longshan Xie, Dongai Yao, Yonghong Liu, Qiang Wu, Song Lan, Kaisheng Liu, Peiyan Zhan, Xiahong Wang, Jingxia Dang, Yunqi Hou, Keqiang Chen, Yulan Zhu, Yuliang Shi, Yunli Yu, Bo Xiao, Suiqiang Zhu, Hongmei Meng

**Affiliations:** ^1^ Department of Neurology Xiangya Hospital Central South University Changsha China; ^2^ National Clinical Research Center for Geriatric Disorders Xiangya Hospital Central South University Changsha China; ^3^ Department of Neurology, Union Hospital, Tongji Medical College Huazhong University of Science and Technology Wuhan China; ^4^ Department of Neurology First Affiliated Hospital of Zhengzhou University Zhengzhou China; ^5^ Department of Neurology, Zhengzhou University People's Hospital Henan Provincial People's Hospital Zhengzhou China; ^6^ Department of Neurology Hubei Provincial Hospital of Integrated Chinese and Western Medicine Wuhan China; ^7^ Department of Neurology Wuhan No. 1 Hospital Wuhan China; ^8^ Department of Neurology Wuhan General Hospital of the YANGTZE River Shipping Wuhan China; ^9^ Department of Functional Neuroscience The First People's Hospital of Foshan Foshan China; ^10^ Department of Neurology Zhongnan Hospital of Wuhan University Wuhan China; ^11^ Department of Neurology Xijing Hospital of Air Force Military Medical University Xi’an China; ^12^ Department of Neurology Wuhan General Hospital of PLA Wuhan China; ^13^ Department of Internal Medicine‐Neurology Maoming People′s Hospital of Guangdong Province Maoming China; ^14^ Department of Neurology Taihe Hospital Shiyan China; ^15^ Department of Neurology The Central Hospital of Wuhan Wuhan China; ^16^ Department of Neurology Zhengzhou Second Hospital Zhengzhou China; ^17^ Department of Neurology The First Affiliated Hospital of Xi’an Jiaotong University Xi’an China; ^18^ Department of Neurology Shunde First Affiliated Hospital of Southern Medical University Shunde China; ^19^ Department of Neurology Central Hospital of Jiangmen Jiangmen China; ^20^ Department of Neurology The Second Affiliated Hospital of Harbin Medical University Harbin China; ^21^ Department of Neurology People's Hospital of Meizhou Meizhou China; ^22^ Department of Neurology Affiliated Hospital of Guizhou Medical University Guiyang China; ^23^ Department of Neurology, Tongji Hospital, Tongji Medical College Huazhong University of Science and Technology Wuhan China; ^24^ Department of Neurology and Neuroscience Center The First Hospital of Jilin University Changchun China

**Keywords:** anxiety and depression, focal epilepsy, levetiracetam, oxcarbazepine, quality of life

## Abstract

**Aims:**

This multicenter, open‐label, randomized study (Registration No. ChiCTR‐OCH‐14004528) aimed to compare the efficacy and effects of oxcarbazepine (OXC) with levetiracetam (LEV) as monotherapies on patient quality of life and mental health for patients with newly diagnosed focal epilepsy from China.

**Methods:**

Patients with newly diagnosed focal epilepsy who had experienced 2 or more unprovoked seizures at greater than a 24‐h interval during the previous year were recruited. Participants were randomly assigned to the OXC group or LEV group. Efficacy, safety, quality of life, and mental health were evaluated over 12‐week and 24‐week periods.

**Results:**

In total, we recruited 271 newly diagnosed patients from 23 centers. Forty‐four patients were excluded before treatment for reasons. The rate of seizure freedom of OXC was significantly superior to that of LEV at 12 weeks and 24 weeks (*p* < 0.05). The quality of life (except for the seizure worry subsection) and anxiety scale scores also showed significant differences from before to after treatment in the OXC and LEV groups.

**Conclusions:**

OXC monotherapy may be more effective than LEV monotherapy in patients with newly diagnosed focal epilepsy. Both OXC and LEV could improve the quality of life and anxiety state in adult patients with focal epilepsy.

## Graphical abstract

Our study reveals 6 months of OXC monotherapy may be more effective than LEV in Chinese patients with newly diagnosed focal epilepsy. We demonstrate that early use of OXC and LEV could improve focal epilepsy patients’ quality of life and anxiety state. However, two drugs may not have a significant effect on patients’ depressive status in the short term.

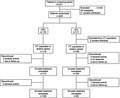

## 1 INTRODUCTION

Epilepsy is one of the most common neurological disorders. Besides epilepsy itself, the improper treatment also affects the prognosis and quality of life of people with epilepsy^1^ and even leads to psychological problems.^2^

Among the new antiseizure medications (ASMs), oxcarbazepine (OXC) and levetiracetam (LEV) are commonly used for patients with focal epilepsy.^3^, ^4^ OXC is a ketoanalogue of carbamazepine (CBZ). As a drug of choice for focal epilepsy in adults and children, OXC has more favorable tolerability and pharmacokinetic profile.^5^ LEV, a broad‐spectrum ASM, may modulate neurotransmission through vesicle protein 2A.^6^ The efficacy and tolerability of LEV are superior to those of other ASMs.^7^ Although many previous studies examining the efficacy of LEV in adult patients with focal epilepsy have been conducted in some Asian countries,^8^, ^9^, ^10^ they investigated LEV as an add‐on therapy rather than a monotherapy. Moreover, many comorbidities, such as anxiety, depression, and other mental disorders, commonly exist in those with epilepsy.^11^, ^12^ Low quality of life of patients with epilepsy is also of great concern in society.^13^ To our knowledge, few studies have compared the effects of ASMs on the quality of life and mental condition between LEV monotherapy and OXC monotherapy in adults with newly diagnosed focal epilepsy.

To expand the available data related to these two ASMs (OXC and LEV) as a monotherapy regarding their efficacy and effects on patients’ quality of life and mental health in Chinese patients with focal epilepsy, we conducted a multicenter, open‐label study, which is the first comprehensive comparison between OXC and LEV for newly diagnosed adult focal epilepsy in China.

## 2 METHODS

### 2.1 Patients and study design

This is a multicenter, open‐label, randomized (1:1) study (ChiCTR‐OCH‐14004528) that investigated the efficacy of OXC monotherapy (900–2400 mg/day) and LEV (1000–3000 mg/day) monotherapy and the effects on patient quality of life and psychological status regarding anxiety and depression. The study was conducted at 23 neurology department centers in China. The ethics committee of each center approved the protocol before patient recruitment commenced. All patients signed written informed consent before participation.

All patients were newly diagnosed with focal epilepsy in accordance with the International League Against Epilepsy classification from 2017,^14^ by epileptologists with abundant clinical experience. All patients had focal aware seizures, focal impaired awareness seizures and/or focal to bilateral tonic‐clonic seizures. Patients aged ≥16 years who had experienced 2 or more unprovoked seizures at greater than a 24‐h interval during the previous year and at least 1 seizure within the last 6 months, without a history of taking any antiseizure medications during the past 6 months, were eligible for inclusion. Patients with weight <40 kg, symptoms, and electroencephalogram (EEG) that indicated idiopathic generalized epilepsy, alcohol abuse in the past 2 years, a history of status epilepticus in the last 3 months, progressive brain disease, such as infection, demyelination, tumors, and degeneration, severe mental or systemic illness, and female patients who were pregnant or breastfeeding were excluded.

**FIGURE 1:**
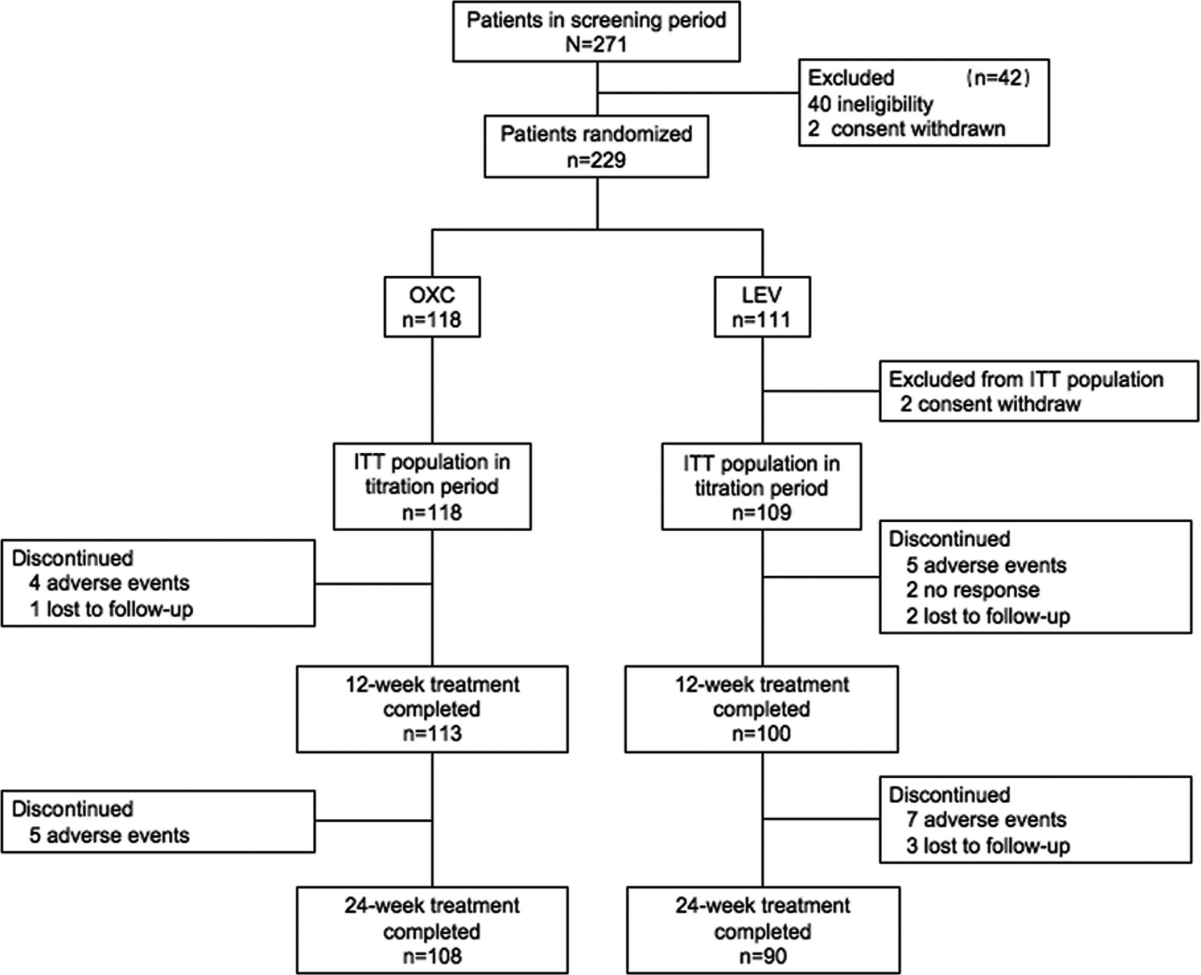
Patient disposition. ITT, intention‐to‐treat; OXC, oxcarbazepine; LEV, levetiracetam

As shown in Figure 1, our study included a 1‐week screening period, after which we initiated intention‐to‐treat (ITT) participants in a 3‐week titration period, during which the patients received either OXC 300 mg/day or LEV 500 mg/day before reaching the first target dosage of 900 mg/day for OXC and 1000 mg/day for LEV. The titration period was followed by a 24‐week treatment and evaluation period. The participants were followed up at 12 weeks and 24 weeks by investigators. If seizures were uncontrolled in the treatment and evaluation periods, dosages of OXC or LEV could be increased up to 2400 mg daily or 3000 mg daily, respectively. All participants could withdraw from the study at any period without confronting any negative consequences.

### 2.2 Assessment

#### 2.2.1 Efficacy and Safety

All patients used daily record cards to record the number of seizures and adverse events (AEs) and reported this information to investigators at 12 weeks and 24 weeks into the treatment and evaluation period. The primary outcome was the rate of seizure freedom, which was defined as the percentage of patients who reached seizure freedom for 12 and 24 successive weeks. The second efficacy outcome was the rate of responders, which was defined as the percentage of patients with a seizure reduction of more than 50% compared with their historical baseline. Moreover, we used the number and percentage of patients with AEs and the discontinuation rate from the study due to AEs as indicators for safety assessment.

#### 2.2.2 Quality of life

Quality of life in epilepsy‐31 (QOLIE‐31) was used to evaluate the quality of life of the participants. Baseline scores were obtained before the titration period, and post‐treatment scores were obtained at 12 and 24 weeks into the treatment and evaluation period. The test was divided into seven subsections: seizure worry, emotional well‐being, energy/fatigue, cognition, medication effects, social function, and overall quality of life. We obtained the subtotal scores for each subsection, and then, the overall test score was derived by weighting and summing the subtotal scores.^15^ Higher scores indicated better quality of life.

#### 2.2.3 Anxiety and depression scale

Anxiety and depression levels were determined using the Self‐rating Anxiety Scale (SAS)^16^ and Self‐rating Depression Scale (SDS).^17^ We obtained baseline scores before the titration period and post‐treatment scores at 12 and 24 weeks into the treatment and evaluation period. If participants had a sum score of greater than 50 on either of the two tests, they were classified as “patients with probable anxiety or depression.”

### 2.3 Statistical analyses

The analyses of efficacy and other parameters, including quality of life, anxiety, and depression, were performed on the ITT basis in the participants who completed the follow‐up evaluation. In our study, the Fisher's exact tests were used for the comparison of efficacy outcomes. The other enumeration data comparisons, such as sex distribution and the number and percentage of patients with AEs, used the Pearson chi‐squared tests or Fisher's exact tests. We used the Shapiro–Wilk tests to analyze the normality of all measurement data. The weight of patients and scores of three scales showed normal distribution. The independent sample *t*‐tests were used to assess the difference in weight between groups. For comparisons of age, frequency of seizures, and duration of epilepsy, the Mann–Whitney U tests were used. Taking the effect of study time points and a random intercept of within‐patient correlation into account, generalized linear mixed models (GLMM) were used to compare scores of three scales. Significant results were reported with *p* < 0.05.

For the calculation of sample size, G*Power 3.1 software was used, and we expected the effect size (effect size = between‐group variance / standard deviation) to be equal to or greater than 0.3. The Type I error rate was set as 0.05, the power as 0.95, and the degree of freedom as 1. The number of ITT patients needed for the two groups was 145.

## 3 RESULTS

### 3.1 Demographic and clinical characteristics

In total, between March 26, 2018, and June 27, 2019, we recruited 271 newly diagnosed patients from 23 neurology department centers. During the screening period, 40 patients were excluded due to idiopathic generalized epilepsy diagnosis and 2 patients withdrew consent. A total of 229 remaining patients, in accordance with the random number table, were divided into the OXC group (*n* = 118) and the LEV group (*n* = 111). Subsequently, two patients withdrew consent in the LEV group, resulting in 118 in the OXC group and 109 in the LEV group enrolled in the ITT group (Figure 1). There were no significant differences in age, sex distribution, weight, frequency of seizures, and duration of epilepsy between the OXC group and the LEV group in the ITT population (*p* > 0.05), as shown in Table 1.

**TABLE 1:** Demographic and focal epileptic characteristics in the ITT population

	OXC group *n* = 118	LEV group *n* = 109	*p*‐value
Age (years), median (IQR)	37 (26–51)	32 (24–45)	0.070
Gender, *n* (%)			
Male	65 (50.9%)	53 (58.4%)	0.403
Female	53 (49.1%)	55 (41.6%)	
Weight (Kg), mean±SD	61.94±9.70	61.39±9.89	0.672
Duration of epilepsy (month), median (IQR)	10.5 (3–50.25)	17 (4–66)	0.396
Numbers of seizures per month, median (IQR)	1 (0.667–2)	1 (0.667–2.333)	0.601

Abbreviations: IQR, interquartile range; ITT, intention‐to‐treat; Kg, kilogram; LEV, levetiracetam; OXC, oxcarbazepine; SD, standard deviation. *p* < 0.05 was accepted as significant.

### 3.2 Efficacy and safety

**FIGURE 2:**
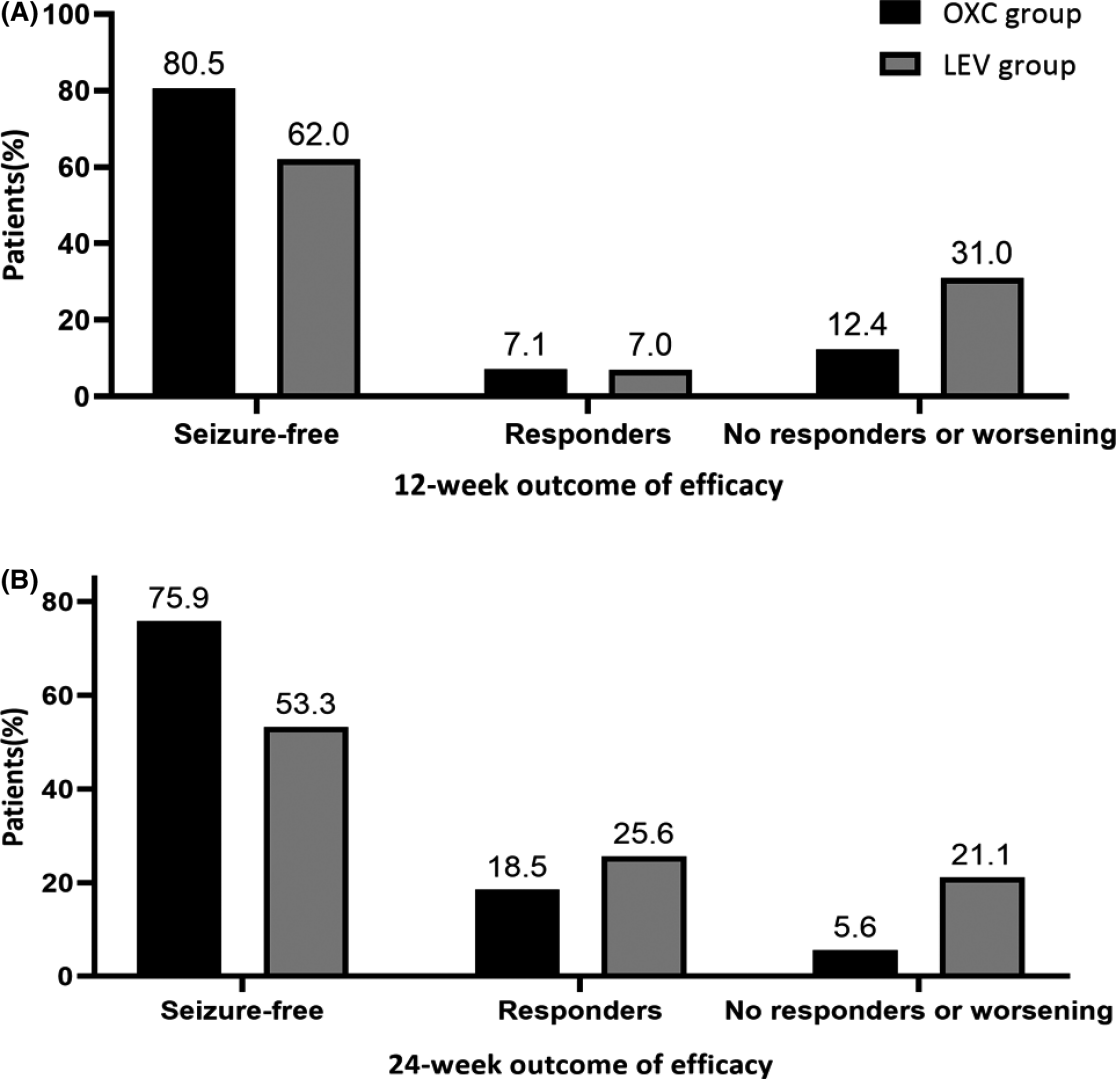
The seizure‐free rate, responder rate (>50% reduction in seizure frequency from baseline), and rate of no response or worsening condition for focal seizures over the 12‐week (A) and 24‐week(B) treatment and evaluation period

Overall, two hundred and thirteen patients completed the 12‐week treatment and evaluation period, and one hundred and ninety‐eight patients completed the 24‐week treatment and evaluation period. The mean doses of OXC and LEV during the treatment and evaluation period were 1031.94±209.86 mg/d and 1225.00±337.39 mg/d, respectively. The number of seizure‐free patients was 91 of 113 (80.5%) in the OXC group, compared with 62 of 100 (62.0%) in the LEV group at 12 weeks into the treatment and evaluation period. Similar results were reported at 24 weeks, 82 of 108 (75.9%) in the OXC group and 48 of 90 (53.3%) in the LEV group, which showed that efficacy in the OXC group was significantly superior to that in the LEV group (*p* < 0.05). The percentages of seizure‐free patients, responders, and patients with no response or worsening condition are shown in Figure 2. The frequencies of seizures of responders and patients with no response or worsening condition in the OXC group were lower than those in the LEV group at 12 weeks and 24 weeks (*p* < 0.05). In the ITT population, 9 (7.6%) patients in the OXC group and 12 (11.0%) patients in the LEV group dropped out of the study due to AEs. Additionally, five participants in the OXC group and four in the LEV group reported that they had experienced adverse events without dropping out of the study. The AEs reported by the participants included dizziness, headache, rash, and somnolence. There were no severe adverse events reported in our study (Table 2). The other reasons for withdrawal included contact loss with participants (1 in the OXC group and 5 in the LEV group) and no response to the drug (2 in the LEV group). The total attrition rate is 8.5% of OXC vs. 17.1% of LEV.

**TABLE 2:** Efficacy and safety of two ASMs after the 12‐week and 24‐week treatment and evaluation period

	OXC group	LEV group	*p*‐value
12‐week efficacy of ASMs, *n*	113	100	
Seizure‐free, *n* (%)	91 (80.5%)	62 (62%)	0.004
Response but not got seizure‐free, *n* (%)	8 (7.1%)	7 (7.0%)	
No response or worsening, *n* (%)	14 (12.4%)	31 (31%)	
12‐week numbers of seizure per month^*^, median (IQR)	1 (0.5–1.125)	1.5(0.875–2.5)	0.032
24‐week efficacy of ASMs, *n*	108	90	
Seizure‐free, *n* (%)	82 (75.9%)	48 (53.3%)	0.001
Response but not got seizure‐free, *n* (%)	20 (18.5%)	23 (25.6%)	
No response or worsening, *n* (%)	6 (5.6%)	19 (21.1)	
24‐week numbers of seizure per month^*^, median (IQR)	0.33 (0.333–0.833)	1 (0.667–1.417)	0.018
Number of drop out study due to AEs, *n* (%)	9 (7.6%)^a^	12 (11.0%)^b^	0.380
Adverse events, *n* (%)	5 (4.6%)	4 (4.4%)	0.613

Note

ASM, antiseizure medication; OXC, oxcarbazepine; LEV, levetiracetam; IQR, interquartile range; AEs, adverse events; ^a^ *n* = 118; ^b^ *n* = 109; ^*^patients who were seizure‐free were excluded from this calculation. *p* < 0.05 was accepted as significant.

### 3.3 Quality of life

**FIGURE 3:**
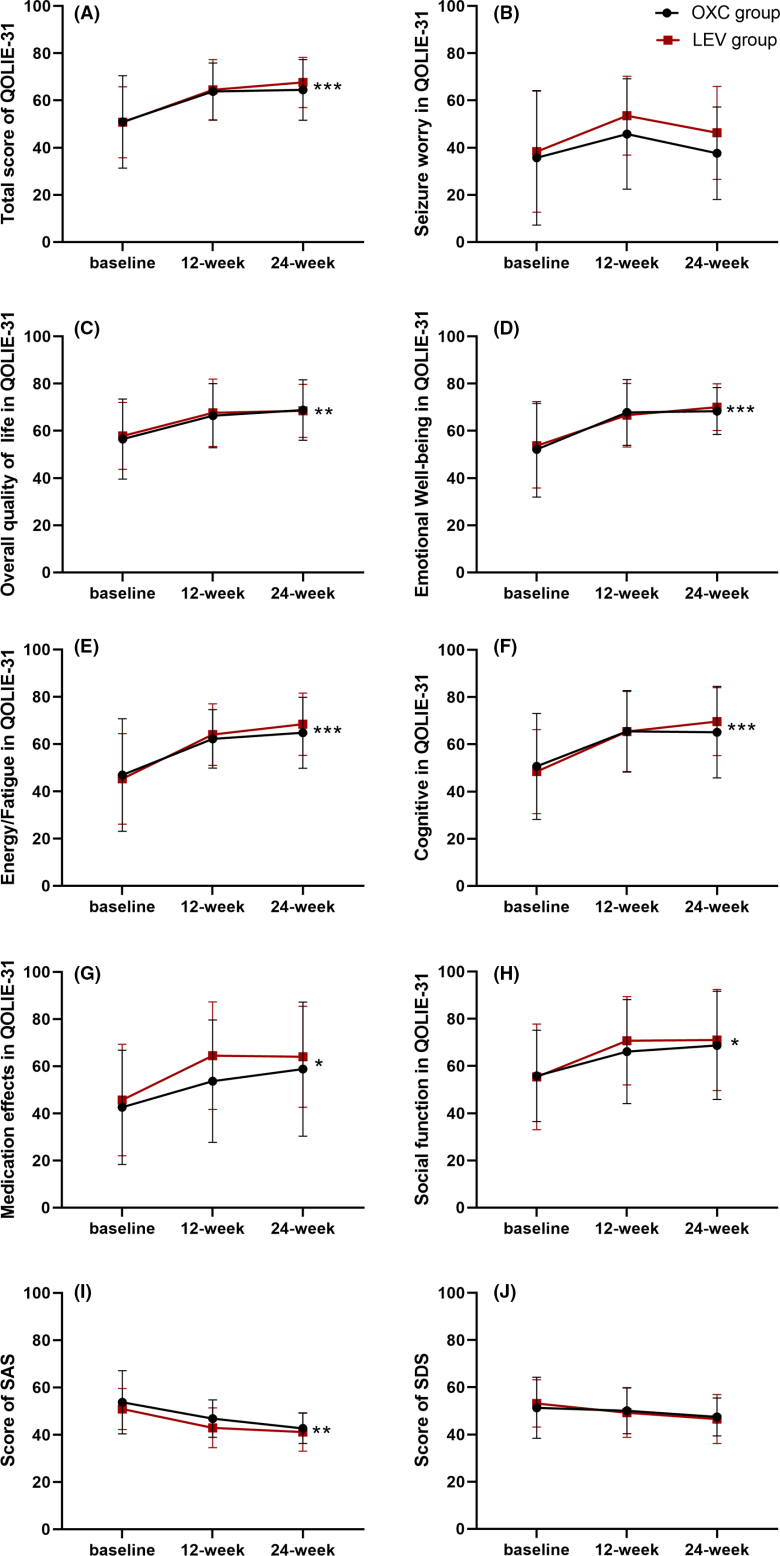
Mean change of QOLIE‐31 (including total score and 7 subsection scores), SAS scores, and SDS scores in two groups. (A) showed the difference of total score of QOLIE‐31 in two groups at each point. (B‐H) showed the difference of the 7 subsections of the QOLIE‐31 in two groups at each point. (I‐J) revealed differences of SAS and SDS in two groups at each point. *p < 0.05, **p < 0.01, ***p < 0.001. OXC, oxcarbazepine; LEV, levetiracetam; QOLIE‐31, Quality of life in epilepsy‐31; SAS, Self‐rating Anxiety Scale; SDS, Self‐rating Depression Scale

The results are shown in Figure 3. The two groups showed significant improvements in the QOLIE‐31 total scores and 6 subsection scores of the QOLIE‐31 (*p* < 0.05) at two points; the exception was no significant difference found in the seizure worry subsection within the OXC or LEV group (*p* = 0.0741). In addition, there was no significant difference between the two groups after treatment (*p* > 0.05).

### 3.4 Anxiety and depression scales

Before treatment with the two drugs, the baseline scores on the neuropsychologic scale assessing anxiety and depression were both greater than 50 points, which decreased to less than 50 points after treatment in both groups. The change in scores was statistically significant for anxiety in the OXC and LEV groups (*p* < 0.05). Although the score on the self‐rating depression scale was reduced after treatment in two groups, the reduction was not statistically significant. Simultaneously, comparison between two groups showed no significant difference after treatment (*p* > 0.05, Figure 3).

## 4 DISCUSSION

This multicenter, open‐label, randomized study was the first comparative study between OXC and LEV as monotherapies in patients with newly adult diagnosed focal epilepsy in China. Using the rate of seizure freedom as the primary outcome assessment, OXC monotherapy was indicated to be superior to LEV in efficacy for the treatment of Chinese adult patients with focal epilepsy. Additionally, the two first‐line new ASMs had similar incidences of AEs (4.6% vs. 4.4%) based on the ITT population who completed all follow‐up evaluations, which demonstrated that both of these ASMs have good tolerability. However, among the ITT population, 9 (7.6%) patients in the OXC group and 12 (11.0%) patients in the LEV group dropped out of the study due to AEs, including dizziness, headache, rash, and somnolence. Although these AEs were generally mild‐to‐moderate in intensity, participants still insisted on withdrawing from the study by changing ASM or reducing drug doses. While there was no statistically significant difference between the two groups (*p* = 0.380), the subtly higher attrition rate in the LEV group appeared to have influenced the final results over the 12‐week and 24‐week treatment and evaluation period.

Regarding the efficacy of the two ASMs, the seizure‐free rates in the OXC and LEV groups in our study were relatively higher than those in previous trials,^18^, ^19^, ^20^, ^21^ which was probably due to our design bias and shorter follow‐up time. Unlike other studies that recruited patients with drug‐resistant epilepsy,^22^ most hospitals participating in our study were primary hospitals where the majority of patients had epilepsy that was mild or moderate with low frequency of seizures, where intractable epilepsy and complications were relatively rare, resulting in a higher seizure‐free rate. Additionally, previous studies have shown that seizure‐free rates were almost identical with LEV and OXC after a 1‐year treatment period.^23^ While in studies with a 3‐year follow‐up, the seizure‐free rate of patients taking LEV increased progressively over time^24^ and the 3‐year rate was significantly better than that with OXC,^25^ which suggested that LEV might be more effective than OXC for the long‐term treatment of newly diagnosed focal epilepsy. In other words, the effect advantage of OXC is reflected in the early stage, but the persistence of efficacy for OXC is not as good as LEV, especially during long‐term monotherapy for epilepsy.

As known, OXC, the formation of an epoxide metabolite that was not involved in biotransformation, provides a compound chemically similar to CBZ to mimic its efficacy and overall tolerability while improving its side‐effect pattern.^26^ Furthermore, as a new broad‐spectrum antiseizure drug, LEV is possibly as effective as OXC in patients with new‐onset focal epilepsy. A meta‐analysis of the efficacy and safety of ASMs for drug‐resistant focal‐onset epilepsy revealed that LEV was associated with a lower study withdrawal rate due to fewer AEs than OXC,^27^ while more patients (12 (11.0%)) in the LEV group withdrew from our trial on the basis of adverse events. As a pyrrolidone derivate compound binding at the vesicle protein 2A receptor site, LEV is active with a unique mode.^28^, ^29^ However, this pharmacological mechanism of LEV also brings about its side effects, especially somnolence, one of the most common reasons for treatment withdrawal.^29^ A subjective feeling of somnolence in patients with epilepsy could affect their daily work and life. When making the ASM treatment plan for newly diagnosed patients in the clinic, in addition to the efficacy of drugs, the side effects are also an aspect we need to take into account. Combined with the efficacy results, OXC may be more effective than LEV in the first 6 months of treatment with fewer side effects in patients with new‐onset adult focal epilepsy.

In addition to the control of seizures, issues of quality of life, social functioning, and mental health in epilepsy should be considered as well, which is also a very important index to evaluate the efficacy of ASM. Whether ASMs could improve the quality of life and mental health of patients with epilepsy greatly depends on if seizures could be controlled.^30^, ^31^ The adverse effects of ASMs have been associated with the incidence of psychiatric or behavioral problems and their severity. The combination results from the three scales we assessed were significantly improved, including the total score and 6 other subsection scores of the QOLIE‐31, covering the overall quality of life, emotional well‐being, energy and fatigue, cognitive, medication effect, and social function. Even with the exception of the seizure worry subsection, the results suggested that quality of life could be improved under the high rate of seizure freedom and respondence, in accordance with previous studies. However, patients still had a greater psychological burden, regardless of whether they will have another seizure, or their seizure control is effective. In other words, they would still be afraid of the next seizure attack. As a previous study showed, anxiety and depression were the most common psychiatric comorbidities in patients with epilepsy.^32^, ^33^ Herein, considering that the participants we recruited were patients with newly diagnosed focal epilepsy, we used the Self‐rating Anxiety Scale and Self‐rating Depression Scale to preliminarily evaluate the mental condition of patients. Set up on the baseline scores (before treatment), the average scores on the anxiety and depression scales were more than 50 points in each group, indicating that they may be prone to anxiety or depression when they were diagnosed with epilepsy. After treatment with OXC or LEV, both anxiety and depression scores dropped to approximately 45 points at 12 weeks and approximately 43 points at 24 weeks into the treatment and evaluation period, and the decline was even greater in the LEV group. Consistent with previous reports in the literature,^34^ when being diagnosed with epilepsy, patients in our study developed a state of anxiety and depression. To some extent, both OXC and LEV could mitigate anxiety and depression after treatment. However, taking OXC or LEV will have many psychiatric side effects in previous studies including suicidal thoughts, aggression, irritability, and cognitive impairment,^35^, ^36^, ^37^ which were reported rarely in our study, indicating that longer follow‐up periods are needed to observe if there are other psychological side effects of these two drugs during treatment.

The present work is still limited in several ways. We acknowledge the open‐label design of our study as the major limitation, which may have biased the statistics in this analysis, and the participants will be subconsciously curious about the differences in efficacy, side effects, and cost of the other group's drug, which could potentially have had an impact on our outcome. Second, patients who experienced at least one seizure in the past 6 months before their participation had the mild or moderate condition, and their follow‐up time was only 12 weeks and 24 weeks in our study, which may lead to overstating the efficacy of two drugs due to the short period of time. A longer follow‐up should be implemented to observe the long‐term response and side effects of OXC and LEV in future work. Third, the substantially higher attrition rate, particularly in the LEV group, could have influenced our results. Finally, we analyzed only Chinese patients, which may limit the application to other ethnic groups of patients.

## 5 CONCLUSION

OXC is probably more effective than LEV monotherapy in treating adult patients with newly diagnosed focal epilepsy. Both OXC and LEV could improve quality of life and anxiety conditions in patients with epilepsy at a 24‐week follow‐up evaluation study.

## CONFLICT OF INTEREST

All authors declare no conflicts of interest in this work. Our study received support from the fund of Novartis China Company, which produces OXC. The sponsor had no role in the design and conduct of the study; collection, management, analysis, and interpretation of the data; or in the preparation and submission of this article.

## Data Availability

Anonymized data will be shared upon request by any qualified investigator.
